# The Treatment of Hemorrhoids

**Published:** 1888-07

**Authors:** Hunter P. Cooper

**Affiliations:** Professor of Chemistry, Atlanta Medical College, Atlanta, Ga.


Vol. v. JULY, 1888. No. 5.

### Original Communications.

### THE TREATMENT OF HEMORRHOIDS.*

BY HUNTER P. COOPER, M. D., ATLANTA, GA.

Professor of Chemistry, Atlanta Medical College, Atlanta, Ga.

The object of this paper is not to consider exhaustively all of the operations which have been devised for the cure of the hemorrhoidal disease, but to discuss from a practical standpoint a few of the best methods. A surgeon in general practice will obtain far better results if he thoroughly familiarizes himself with the details of two or three operations than he will by attempting to perform all of the dozen or more which have been recommended.

Hemorrhoids are naturally divided into the external and internal.

The external hemorrhoids are of far less surgical importance than the internal, and yet they are of such frequent occurrence, and are a source of so great annoyance, that it may not be out of place to say a few words about them.

*Read before the Medical Association of Georgia, April, 1888.

A correct appreciation of the pathology of the different varieties of external hemorrhoids is absolutely essential if we expect to treat them successfully. The first variety is that form in which a small swelling appears suddenly at the anal margin, which is hard, excessively tender to the touch and bluish in color. The patient cannot walk or sit down with any comfort; there is a sense of fullness and throbbing in the lower part of the rectum. At first the patient finds great relief in the recumbent position, but as the swelling increases in size, the patient is often unable to lie down with comfort. In those of nervous temperament, notably in women, sleep is often very much broken by painful, spasmodic contractions of the sphincter muscle. In its worst form, the patient is often confined to his bed for a number of days and does not get entirely well for nearly two weeks.

The cause of this form of hemorrhoid is the rupture of a small branch of the inferoir hemorrhoidal vein, and the escape of the blood into the surrounding tissues. Here it excites inflammation, which causes the parts to swell and become very painful. With recovery the swelling gradually disappears entirely, or else becomes converted into a painless, cutaneous tab.

This cutaneous tab forms the second variety of the external hemorrhoid. Under ordinary circumstances it remains painless, and the patient is hardly aware of its existence. It is simply an hypertrophy of the skin at the margin of the anus. When appropriate conditions are present, however, it takes on acute inflammation, becomes very painful and swollen and gives rise to the same set of symptoms as the first variety.

The third variety of external hemorrhoids is one that I have not seen described by any author, though I suppose that circumstance is due to my rather limited library. In this variety there it *no* rupture of a vein with the formation of a hard, painful swelling, and there is *no* cutaneous tab. Ocular examination of the anus reveals a rather varicose condition of one of the veins. Pressure on this vein with the finger demonstrates the fact that there is no hardness whatever, though considerable pain is elicited. The symptoms of this variety are the same as in the other forms, though the disability is not so marked. I am particular about insisting on such a division of external hemorrhoids, because the treatment in each variety is entirely different.

The treatment of piles has brought down considerable reproach on the medical profession, and given rise to dozens of patent pile ointments and hordes of pile quacks. The day of treating all cases of hemorrhoids by astringent and anodyne ointments without so much as even examining the patient has gone by. Every patient presenting the symptoms of external hemorrhoids should be carefully examined in order that a correct diagnosis may be made. If the patient will not submit to an ocular, substitute a digital examination. Such an examination will often develop the fact that the patient is not suffering from hemorrhoids at all, but from fistula or fissure. The treatment of the different varieties of external hemorrhoids is very simple and effective. In the first variety, where the rupture of a small vein has led to the formation of a hard, bluish, painful tumor at the margin of the anus, the treatment consists in laying open the swelling with a sharp bistoury and turning out the clot. The venous oozing should be ehecked before leaving the patient; this is easily accomplished by placing over the wound a small compress, and over this a folded towel, then directing the patient to straddle the back of a chair, and thus make pressure upon the spot for ten or fifteen minutes. A simple dressing with some soothing ointment constitutes the after-treatment, and the patient is well in from 24 to 48 hours.

If the patient objects to even so simple an operation as this (and such is often the case), we must resort to some other means. Under these circumstances nothing is so grateful and efficacious as the local application of ice. The best mode of applying ice to the anal region is to crush it well in a towel, fill an ordinary rubber condom (corrupted into cundrum) with it, close its mouth with a string, put it lengthwise in the gluteal furrow and hold it in place with a T bandage. The cold diminishes the inflammation better than any other agent, and at the same time lessens the tendency to sphincteric spasm. In addition to this an anodyne ointment should be constantly applied to the parts in order to keep the skin soft. The bowels should of course be kept open by laxatives which are gentle in their action.

If we can prevail on the patient to submit to the incision of the swelling, however, the treatment is greatly shortened.

In the second variety, where the symptoms depend upon inflammation of a pendulous tab of skin at the margin of the anus, the treatment is rapidly curative. The cutaneous swelling is seized with a pair of forceps and snipped off with a pair of sharp scissors. Very little bleeding follows this; a simple ointment is applied and the patient is soon well. In this form pain can be abolished by injecting a few drops of a 5 per cent. solution of cocaine into the swelling before cutting it off.

In the third variety, where there is a varicose condition of a small vein at the margin of the anus, the symptoms depend, I think, upon two causes: 1st, an obstruction to the flow of the blood through the vein; 2d, more or less inflammation in the coat of the vein.

Examination with the finger in these cases will reveal an unusually tight condition of the internal sphincter. This contraction prevents the free return of the blood through the vein and keeps up the trouble. The treatment hinges upon this fact—stretch the internal sphincter daily by means of an appropriate rectal speculum and patient is cured in a few days. The result follows very quickly. In one case I treated, the patient had suffered for several days great pain in walking and sitting. The first stretching gave complete relief for two days. The symptoms then returned slightly, and it required two more stretchings to effect a cure. This method is simple and invaluable. The speculum should be very gently introduced and its blades gradually opened. The stretching causes considerable pain, lasting sometimes as much as an hour, but after that great relief is experienced.

Internal hemorrhoids are of vastly greater importance from a surgical standpoint than the external. They are likewise divided into three varieties: 1st. The small, bright red hemorrhoid, called the capillary hemorrhoid. It projects but little above the surrounding surface, and consists of a number of enlarged and interlacing capillaries. This is the form which bleeds so profusely. It is generally easy to cure by applying strong nitric acid to its surface. 2d. The venous internal hemorrhoids. These consist of large, soft, venous tumors, which often come down during a straining effort. When reduced to their natural position in the rectum they can hardly be felt by the examining finger. 3d. The arterial internal hemorrhoid is a tumor in which arterial blood predominates. They are of much firmer consistence than the venous form, and are fed by a pretty large arterial branch.

For the treatment of venous and arterial hemorrhoids many operations have been devised. No less than twelve are enumerated by Allingham. Each of these operations has strong advocates, but it is very important to bear in mind the fact that no single one of these operations is applicable to all cases. Most of these methods I have no personal experience with, so I will confine my remarks to the following:

1st. Verneuil's method of dilatation.

2d. Hypodermic injection of carbolic acid.

3d. Allingham's method by ligature.

1. Dilatation as a method of treatment for internal hemorrhoids was discovered by Verneuil apparently by accident, and is undoubtedly curative in the class of cases to which it is adapted. It commends itself to the laity because it has so little of the appearance of an operation about it. It is indicated in cases where the venous element predominates in the hemorrhoid, and where for any reason a radical operation cannot be done. It is never employed in the capillary hemorrhoid and rarely in well-marked arterial tumors.

The dilatation of the sphincter may be done either by the rectal divulsor, or by the hands of the operator. In the latter case, the two thumbs are inserted into the anus and then forcibly separated. This stretching is continued until the sphincter gives way, which is known to be the case when the resistance ceases. In very muscular subjects it often requires a greater amount of force to rupture the sphincter than can be exerted by the thumbs. In such cases I use the rectal divulsor of Thebaud.

After the operation the patient should be kept in bed until the sphincter has regained its power. Sometimes this paralysis of the lower end of the gut remains permanent, causing incontinence of feces. Such an unfortunate result is more liable to occur in women than in men. In view of the possibility of such an accident, we should use great care in dilating where the sphincter is naturally weak.

2. Injection of carbolic acid.

This operation was practiced for a long time by quacks before the regular profession adopted it.

At first all were carried away with enthusiasm by the simplicity and efficacy of the method. By and by, however, the reaction set in and numerous failures began to be reported. These failures were often attended by disastrous accidents, such as great pain, sloughing, ulceration, abscesses and fistula. Kelsey, of New York, who in 1885 made a glowing report of nearly 200 cases in the *American Journal of Medical Sciences*, says, in 1887: "In writing now I shall use less glowing terms than I did then, but I have by no means abandoned the practice." And again, "All went well for a time, but soon I began to be troubled with a constant succession of sloughs with their attendant pain, and the worst of the trouble was that I never knew beforehand when a slough was likely to be caused." [The Diagnosis and Treatment of Hemorrhoids. By Chas. B. Kelsey, M. D. New York. Geo. S. Davis, Detroit, 1887.]

I, myself, was called to a case in December last, in which the attending physician had injected an internal hemorrhoid with carbolic acid. He had performed the injection carefully and skillfully, but great pain followed, the hemorrhoid injected and the one adjacent became acutely inflamed. I applied a ligature to each of them, cut them off and the patient got well without a bad symptom.

In another case in my practice the injection was followed by a painful ulcer.

It is undoubtedly true, on the other hand, that many cases are painlessly and permanently cured without confining the patient to bed, and without subjecting him to the terrors of a cutting operation. Good results are more apt to follow this treatment when the hemorrhoid is arterial in character. The technique of the operation is very simple. A solution of pure carbolic acid in glycerine or sperm oil is made of a strength varying from 10 to 50 per cent. With an ordinary hypodermic syringe five minims of the solution are injected into the middle of the hemorrhoid. The tumor is then replaced and the patient allowed to go about his business. Sometimes one injection causes the tumor to disappear. In other cases it is necessary to repeat the injection once or twice.

Before considering the operation by ligature, I will make brief mention of two other operations: 1st, Pollock's operation of crushing; 2d, Henry Smith's operation with the clamp and cautery. Pollock's operation is performed by an instrument called the screw-crusher, by the means of which the hemorrhoidal tumor is reduced to a pulpy mass. Of this operation Allingham says in the *International Encyclopedia of Surgery:* "In over 500 cases treated by crushing, I have not had one death from any cause whatever."

Henry Smith's operation with the clamp and cautery has been warmly advocated in this country by Kelsey, of New York. I have had no experience whatever with it, but on theoretical principles it would seem inferior to the ligature.

3d. The ligature.

The treatment of internal hemorrhoids by the ligature was brought to its present perfection by Allingham, of London, though it was originated by Salmon. This operation has stood the test of over forty years, and is to-day probably performed oftener than all the other oparations put together. As to its safety, Allingham has had only one death in 1,800 cases of ligation. (*International Encyclopedia of Surgery, Vol. VI. p. 132.*)

Gross says: "The operation is as simple of execution as it is free from danger and certain in its results."

Van Buren says, speaking of this operation, "I have never had an unpleasant symptom." Bodenhamer says, "I have yet to encounter my first serious accident." (Quoted from Allingham's "Diseases of the Rectum.")

A private letter from Dr. Sands, of New York, states that it is the operation preferred in New York.

And yet, in the face of such opinions from such men, we find objections to the ligature. These objections are often urged by those who do not perform the operation as advised by Allingham. A patient, who is to be subjected to this operation, should have the bowels freely acted upon for two days preceding the operation, either by calomel or castor oil. On the day appointed a copious enema of warm water and soap-suds will complete the work of clearing out the bowels.

After etherizing the patient the surgeon should always thoroughly divulse the sphincter, either with his thumbs or Thebaud's rectal divulsor. This is a step in the operation that cannot be too strongly insisted on. As has been pointed out, the object of this thorough stretching is two-fold. In the first place it gives the surgeon perfect command of the field of operation. In the second place it prevents the painful spasmodic contractions of the sphincter which take place if the sphincter is not thus thoroughly paralyzed. The next step is to seize the hemorrhoid with a vulsellum and draw it down. Then, with a pair of sharp scissors, a furrow is cut in the mucous membrane all around its base if the tumor be chiefly venous. If it is arterial, the lower part of the base can be pretty freely cut away from its attachments to the gut, for the arterial branch supplying it enters at its upper part. If the hemorrhoid has been long prolapsed, and has a partial cutaneous covering, the surgeon should be careful to separate it from the skin with the scissors so as to include as little skin as possible in his ligature. A ligature of strong antiseptic silk is next passed around the tumor and tightly tied in a triple knot. The hemorrhoid is then cut off, leaving sufficient stump as security against slipping of the ligature.

The rectum should then be thoroughly washed out by a 1 to 1,000 solution of bichloride of mercury, a little iodoform ointment (with vaseline) smeared over the cut surfaces, a thick compress of antiseptic gauze placed against the anus, held in place by a snug T bandage, and the patient put to bed. I am in the habit of keeping the bowels locked with opium for one full week, during which time the dressing is changed several times, and after the third day traction is gently made on the cut ends of the ligatures. At the end of the 6th day I order a large dose of castor oil and the following morning an enema of oil and warm water. This usually causes considerable pain, but subsequent movements of the bowels are accomplished with ease and the patient is well.

Now as to some of the objections to the operation, and I have done. Kelsey, who advocates the clamp and cautery operation, says the following concerning the *ligature:* "It is as safe as any operation can well be, and, when properly done, it cannot fail to cure." After paying it this high compliment, he objects to it because of the great pain the patients suffer for a week after the operation.

With the vast majority of operators complete *absence* of pain is one of the strong points in favor of the operation. I am sure that if Allingham's method is faithfully carried out as to divulsion of the sphincter and cutting away of the tissues forming the lower part of the base of the hemorrhoid, pain will be but rarely encountered. In my own experience one of the chief charms about the operation by ligature is the freedom from pain. The other objections urged by Kelsey are, the necessity of drawing off the urine for several days, the amount of blood lost during the operation, and the length of time (one week) required for a cure.

These objections "vanish into thin air" when compared with the great advantage which the ligature has over the clamp and cautery, viz., the far greater certainty of not having hemorrhage *after* the operation. Which is the more certain safe-guard against bleeding from a cut vessel: a stout piece of silk tied tightly around it or a thin crust of charred tissue?

